# Association of the S2 allele of the *Sst*I polymorphism in the *apoC3* gene with plasma apoCIII interacts with unfavorable lipid profiles to contribute to atherosclerosis in the Li ethnic group in China

**DOI:** 10.1186/s12944-017-0614-3

**Published:** 2017-11-21

**Authors:** Minzeng Sun, Lin Chen, Hui Liu, Lihui Ma, Tiansong Wang, Yueli Liu

**Affiliations:** 1 Department of Pharmacology, School of Pharmacy, Hainan Medical University, Haikou, Hainan Province 571199 China; 2 Department of Cardiology, People’s Hospital of Sanya, Sanya, Hainan Province 572000 China; 3 Department of Anatomy, School of Basic Medicine and Life Sciences, Hainan Medical University, Haikou, Hainan Province 571199 China

**Keywords:** S2 allele, ApoCIII, Atherosclerosis

## Abstract

**Background:**

The *Sst*I polymorphism in the apolipoprotein 3 gene (*apoC3*) has been identified in many ethnic groups. In addition, the S2 allele of the SstI polymorphism is shown to be associated with increased plasma triglyceride (TG) levels. Plasma apoCIII is an important atherogenic factor, which interrupts lipid metabolism and is positively associated with plasma TG levels. However, the existence of the *Sst*I polymorphism in the Li ethnic group in China remains to be confirmed. The relationship between the S2 allele of the *Sst*I polymorphism and plasma apoCIII or TG and their roles in atherosclerosis are also unknown.

**Methods:**

A cohort of 628 participants was recruited (316 atherosclerotic patients and 312 healthy controls) from both the Li and Han ethnic groups. Blood samples were obtained to evaluate the *Sst*I polymorphism in the *apoC3* and lipid profiles. Chi-squared and *t*-tests and multiple unconditional logistic regression were employed to analyze the genotypic and allelic frequencies and lipid profiles using SPSS version 20.0 software.

**Results:**

The *Sst*I polymorphism in the *apoC3* was identified in the Li ethnic group. The S2 allele and plasma apoCIII and TG levels were associated with the development of atherosclerosis (*P* < 0.01, S2 allele and apoCIII; *P* < 0.05, TG) in the Li ethnic group. The S2 allele was associated with increased plasma apoCIII levels in the atherosclerotic group (*P* < 0.01), but with increased plasma apoCIII and TG levels in control group (both *P* < 0.01). In addition to the increases in the S2 allele frequency and plasma TG and apoCIII levels, atherosclerotic patients in the Li ethnic group also exhibited increased apoB, decreased HDL-C and apoAI and a lower apoAI:apoB ratio (all *P* < 0.01).

**Conclusions:**

Our results indicate that the S2 allele of the *Sst*I polymorphism in the *apoC3* gene is associated with plasma apoCIII levels in the Li population. In combination with unfavorable lipid profiles, this might contribute to susceptibility to atherosclerosis.

## Background

Several polymorphisms of the apolipoprotein C3 gene (*apoC3*) have been reported, the *Sst*I polymorphism being the most well-known [1–3]. This polymorphism is defined as a C to G conversion in the 3′ untranslated region of exon 4 in the *apoC3* [4], and has been confirmed to exist in several ethnic groups, including African American, Caucasian, Taiwanese and Chinese populations. There are two alleles of the *Sst*I polymorphism: S1 and S2. Several studies have shown that the rare S2 allele is associated with increased plasma triglyceride (TG) levels, although this association is not always concordant among different ethnic groups [5–7].

The Li population is the smallest minority ethnic group in China, who reside predominantly in Hainan Province, which is not part of Mainland China. However, the existence of the *Sst*I polymorphism in the *apoC3* and the relationship between the S2 allele and plasma TG remain to be confirmed in the Li ethnic group.

Plasma TG is an atherogenic factor that plays a crucial role in the development of atherosclerosis [8–10]. Previous studies have shown that plasma apoCIII is also an independent risk factor for atherosclerosis, and the suppression of apoCIII is associated with decreased risks of atherosclerosis and coronary heart disease (CHD) [11–14]. Plasma apoCIII is also associated with plasma TG [3, 4, 6, 7]; however, the effects of the S2 allele on the risk of atherosclerosis and the relationships between the S2 allele and plasma levels of TG or apoCIII remain to be clarified in the Li ethnic group.

In this study, we investigated the existence of the *Sst*I polymorphism in the *apoC3* in the Li ethnic group. We also investigated the relationships between the S2 allele and plasma TG or apoCIII levels and their roles in the development of atherosclerosis.

## Methods

### Study population

This study was approved by the Ethics Committee of the First Affiliated Hospital of Hainan Medical University (Hainan Province, China). Written informed consent was obtained from all subjects and the investigations were carried out in accordance with the principles of the Declaration of Helsinki. From September 2012 to September 2014, a total of 628 subjects were selected randomly from the Li and Han populations to attend our study. This group included 316 atherosclerotic subjects (AS), consisting of 155 Li individuals and 161 Han individuals with an average age of (63.68 ± 12.19 y). In addition, 312 healthy subjects (control) were selected consisting of 158 Li individuals and 154 Han individuals with an average age of (61.75 ± 13.07 y). The age range of the participants was between 41 and 88 y. There was no consanguinity among the participants, whose grandparents and parents were from the same population and resided in Hainan Province. Atherosclerotic subjects were determined by angiographic or ultrasonic detection of atherosclerotic plaques in the carotid or coronary arteries. Patients with a history of inflammatory disease, autoimmune disease, malignant tumors, familial hyperlipidemia or diabetes were excluded. Healthy subjects were screened on the basis of having no history of hypertension, cardiovascular or cerebral disease, and endocrine disease.

### Epidemiological survey

An international standard method was applied to survey demographics, socioeconomic status, and lifestyle using a standardized questionnaire to gather and record all information. Smoking status and alcohol status were categorized into two groups: non-smoker and smoker, and non-drinker and drinker. The physical examination included measurements of body height, body weight, waist circumference, blood pressure, electrocardiograms, and body mass index (BMI). Blood pressure referred to the average sitting blood pressure after three measurements made using a mercurial sphygmomanometer. Systolic and diastolic blood pressures referred to the first and the fifth Korotkoff sounds, respectively. BMI was calculated by dividing weight (kg) by squared height (m^2^). Abdominal obesity was defined as waist circumference ≥ 90 cm for males and ≥85 cm for females. Overweight was defined as BMI ≥25 kg/m^2^, and hypertension was diagnosed according to diagnostic criteria based on Chinese Guidelines for Prevention and Treatment of Dyslipidemia in Adults and the 2010 Chinese Guidelines for Prevention and Treatment of Hypertension.

### Biochemical analysis

Venous fasting blood samples (10 ml) were obtained after a 12 h overnight fast. From this sample, 6 ml was used for analysis of the serum levels of TG, total cholesterol (TC), low density lipoprotein cholesterol (LDL-C), high density lipoprotein cholesterol (HDL-C), lipoprotein AI (apoAI), lipoprotein B (apoB), apoCIII, lipoprotein a [Lp(a)] and fasting blood sugar (FBS). The remaining 4 ml was placed into a tube coated with the anticoagulant ethylene diamine tetra-acetic acid (EDTA) for extraction of deoxyribonucleic acid (DNA). TG, TC, HDL-C, LDL-C, and FBS levels were determined using enzymatic methods; and apoAI, apoB and Lp(a) were assessed using immunoturbidimetric assays with commercially available kits according to the manufacturer’s instructions (Pointe Biotech, China). ApoCIII was measured by enzyme-linked immunosorbent assay (Abcam, Cambridge, England). All samples were processed using an autoanalyzer (Type 7600; Hitachi Ltd., Tokyo, Japan) or ELISA plate reader (Biotech, USA) at the First Affiliated Hospital of Hainan Medical University.

### DNA amplification and genotyping

DNA was extracted from leukocytes present in the anti-coagulant-treated blood sample using a DNA extracting kit (Sigma-Aldrich, USA). Restriction fragment length polymorphism (RFLP) analysis was employed to detect the *Sst*I polymorphism in the *apoC3* gene. The *apoC3* gene was amplified by polymerase chain reaction (PCR) using the following primers: forward 5′-CATGGTTGCCTACAGAGGAGTTC-3′ and reverse 5′-TTTGACCTTCCGCACAAAGCTGT-3′ (Sangon, Shanghai, China) in a total volume containing: 10 μl (300 ng) of template DNA, 2 μl of each primer, 13.5 μl of 2× EcoTaq PCR SuperMix, and 22.5 μl of RNase-Free water. The amplification process was optimized using a T Gradient Thermocycler (Biometra, Germany) with an initial denaturation at 94 °C for 2 min, followed by 35 cycles of denaturation at 95 °C for 1 min, annealing at 56.2 °C for 45 s, and extension at 72 °C for 1 min, with a final extension at 72 °C for 10 min. The amplification products were then subjected to RFLP analysis in a total reaction volume of 25 μl consisting of 10 μl of amplified DNA, 2.5 μl of 10× NEB buffer, 1 μl of *Sst*I restriction enzyme and 11.5 μl of ddH_2_O at 37 °C for 4 h.

### DNA sequencing

The S1S1, S1S2 and S2S2 genotypes identified by PCR-RFLP were also verified by DNA sequencing of both strands using the BigDye Terminator v3.1 Cycle Sequencing Kit (Applied Biosystems, Forster City, CA, USA) and an ABI 3730xl DNA Analyzer (Applied Biosystems).

### Statistical analysis

Statistical analysis was carried out using SPSS version 20.0 software, and data were presented as mean ± standard deviation (SD). Quantitative values were analyzed by *t*-test. Observed genotypic frequencies were compared to expected genotypic frequencies to assess Hardy–Weinberg equilibrium. The allele frequency of the *Sst*I polymorphism in the *apoC3* was determined by gene counting, and the chi-squared test was employed to test differences between the genotypic and allelic frequencies of the atherosclerotic patients and healthy controls in both the Li and Han ethnic groups. Differences in demographic characteristics were assessed by the chi-squared or *t-*tests. Multiple unconditional logistic regression analysis was employed to evaluate the effects of the *Sst*I polymorphism in the *apoC3* on the risk of atherosclerosis. Differences in the lipid profiles among the different genotypes were evaluated by *t-*test (intergroup comparisons of serum lipid profiles were performed with the Kruskal–Wallis test or the Wilcoxon–Mann–Whitney test). *P*-values <0.05 were considered to indicate statistical significance.

## Results

### Demographic characteristics of atherosclerotic patients and healthy subjects

There were no significant differences between the Li and Han populations included in the study in terms of the male:female ratio, mean age, height and body weight, BMI, smoking status and drinking status (all *P* > 0.05). Atherosclerotic subjects exhibited greater waist circumference and higher systolic and diastolic blood pressures than healthy subjects (*P* < 0.01, waist circumference and systolic pressure; *P* < 0.05, diastolic pressure) (Table 1).

**Table 1:** Demographic characteristics of atherosclerotic patients (AS) and healthy controls (control) in the Li and Han ethnic groups

Characteristic	AS (*n* = 316)	Control (*n* = 312)	*t (χ* ^*2*^ *)*	*P-*value
Nationality (Li/Han)	155/161	158/154	0.159	0.690
Sex (male/female)	176/140	150/162	3.651	0.056
Age (years)	63.68 ± 12.19	61.75 ± 13.07	1.908	0.057
Height (m)	1.65 ± 0.06	1.64 ± 0.06	1.778	0.076
Weight (kg)	65.49 ± 7.35	64.61 ± 7.32	1.511	0.131
BMI (kg/m^2^)	24.12 ± 2.24	24.06 ± 2.33	0.313	0.755
Non-smoker/smoker	217/99	235/77	3.441	0.064
Non-drinker/drinker	255/61	232/80	3.621	0.057
Waist circumference (cm)	83.14 ± 3.94**	75.34 ± 5.41	20.618	0.000
Systolic pressure (mmHg)	139.85 ± 22.05**	133.27 ± 11.51	4.698	0.000
Diastolic pressure (mmHg)	81.93 ± 11.65*	80.28 ± 7.35	2.118	0.035

Data represent mean ± SD. BMI: Body mass index, ^*^ *P* < 0.05, ^**^ *P* < 0.01, atherosclerotic patients versus healthy controls analyzed using *χ* ^*2*^-tests or *t*-tests

### Determination of the SstI polymorphism in the apoC3 gene

**Fig. 1:**
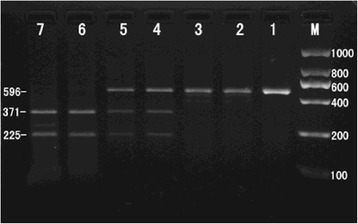
Genotyping of the *Sst*I polymorphism in the *apoC3* gene by RFLP of PCR amplification products. Lane M, 2000 bp ladder marker; Lane 1, PCR product amplified from blood samples (596 bp); Lanes 2 and 3, S1S1 genotype (596 bp); Lanes 4 and 5, S1S2 genotype (596 bp, 371 bp, 225 bp); Lanes 6 and 7, S2S2 genotype (371 bp, 225 bp)

**Fig. 2:**
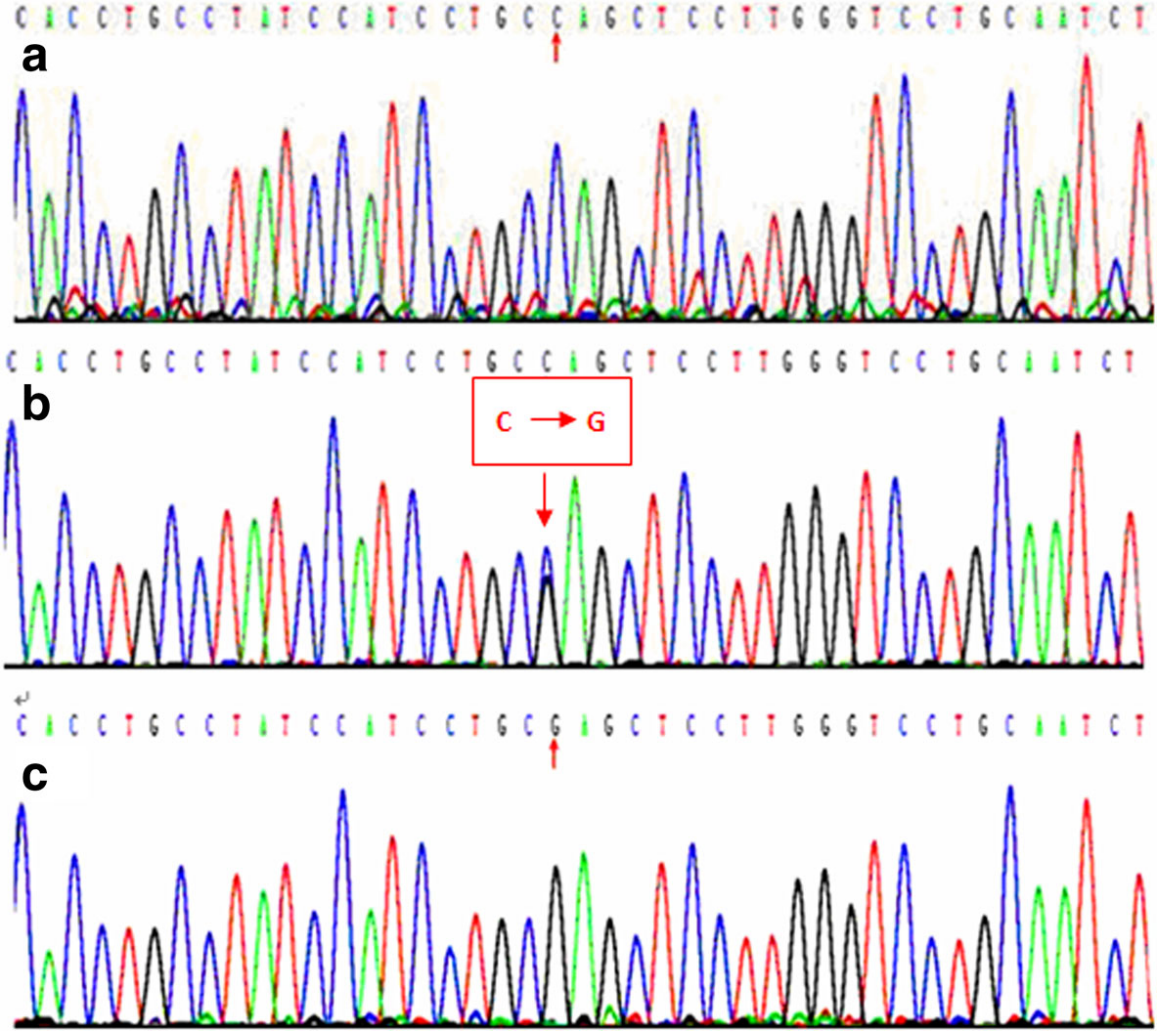
Genotyping of the *Sst*I polymorphism in the *apoC3* gene by DNA sequencing. **a** S1S1 genotype (CC); **b** S1S2 genotype (CG); **c** S2S2 genotype (GG)

Samples were genotyped on the basis of *Sst*I RFLP analysis of PCR amplification products (Fig. 1). A single 596 bp fragment indicated homozygosity for the absence of *Sst*I restriction sites; this was defined as the S1S1 genotype (CC). Fragments of 596 bp, 371 bp and 225 bp indicated heterozygosity for the presence and absence of *Sst*I restriction sites; this was defined as the S1S2 genotype (CG). Fragments of 371 bp and 225 bp indicated homozygosity for the presence of *Sst*I restriction sites; this was defined as the S2S2 genotype (GG). Genotypes were confirmed by DNA sequencing (Fig. 2).

### Genotypic and allelic frequencies in atherosclerotic patients and healthy controls

The genotype frequencies of the atherosclerotic patients and healthy controls were in Hardy–Weinberg equilibrium (*P* > 0.05) (Table 2). However, there were significantly higher frequencies of the S1S2 and S2S2 genotypes among the atherosclerotic patients compared with those among the healthy controls (both *P* < 0.01). Furthermore, the frequency of the S2 allele was higher in atherosclerotic patients compared with that in the healthy controls (*P* < 0.01) (Table 3). Further analysis revealed identical patterns in the Li and Han ethnic groups (Table 4).

**Table 2:** Hardy–Weinberg equilibrium of the genotype distributions of atherosclerotic patients (AS) and healthy controls (control)

Group		Frequency of different genotypes (n, %)			*χ* ^*2*^	*P*-value
		S1S1	S1S2	S2S2		
AS	Actual frequency	165 (52.2)	129 (40.8)	22 (7.0)	0.070	0.966
	Theoretical frequency	167 (52.8)	126 (39.9)	23 (7.3)		
Control	Actual frequency	239 (76.6)	64 (20.5)	9 (2.9)	0.997	0.608
	Theoretical frequency	235 (75.3)	71 (22.8)	6 (1.9)		

Data are expressed as numbers and percentages. Actual frequency versus theoretical frequency within atherosclerotic patients and healthy controls analyzed using *χ* ^*2*^ tests

**Table 3:** Genotypic and allelic frequencies among atherosclerotic patients (AS) and healthy controls (control)

Group	n	Genotypic frequencies(n, %)			Allelic frequencies (n, %)	
		S1S1	S1S2	S2S2	S1	S2
AS	316	165 (52.2)	129 (40.8) ^**^	22 (7.0) ^**^	459 (72.6)	173 (27.4) ^**^
Control	312	239 (76.6)	64 (20.5)	9 (2.9)	542 (86.9)	82 (13.1)
*χ* ^*2*^		40.873			19.734	
*P-*value		0.000			0.000	

Data are expressed as numbers and percentages. ^**^ *P* < 0.01, atherosclerotic patients versus healthy controls analyzed using *χ* ^*2*^ tests

**Table 4:** Genotypic and allelic frequencies in the Li and Han ethnic groups

Ethnic group	Group	n	Genotypic frequencies (n, %)			Allelic frequencies (n, %)	
			S1S1	S1S2	S2S2	S1	S2
Li	AS	155	82 (52.9)	67 (43.2) ^**^	6 (3.9) ^**^	231 (74.5)	79 (25.5) ^**^
	Control	158	122 (77.2)	33 (20.9)	3 (1.9)	277 (87.7)	39 (12.3)
	*χ* ^*2*^		20.376			17.699	
	*P*-value		0.000			0.000	
Han	AS	161	83 (51.6)	62 (38.5) ^**^	16 (9.9) ^**^	228 (70.8)	94 (29.2) ^**^
	Control	154	117 (76.0)	31 (20.1)	6 (3.9)	265 (86.0)	43 (14.0)
	*χ* ^*2*^		20.513			21.462	
	*P*-value		0.000			0.000	

Data are expressed as numbers and percentages. ^**^ *P* < 0.01, atherosclerotic patients versus healthy controls in the Li and Han ethnic groups analyzed using *χ* ^*2*^ tests

### Multiple unconditional logistic regression analysis of the effects of SStI polymorphism in the apoC3 gene on the risk of atherosclerosis

In multiple unconditional logistic regression analysis, atherosclerosis was used as the dependent variable (atherosclerosis: yes = 1, no = 0), while the independent variables were as follows: age (≥50 = 1,<50 = 0), smoking (yes = 1, no = 0), drinking (yes = 1, no = 0), BMI (≥25 = 1,<25 = 0), *apoC3* genotype (S1S1 = 0, S1S2 or S2S2 = 1). According to the results, the S1S2 and S2S2 *apoC3* genotypes were associated with a 2-fold increase in the risk of atherosclerosis (Table 5), indicating that the S2 allele contributes to susceptibility to atherosclerosis.

**Table 5:** The effects of *SSt*I polymorphism in the *apoC3* gene on the risk of atherosclerosis

Variable	AS (n)	Control (n)	Adjusted OR	95% CI	Adjusted *P*-value
Li ethnicity					
*ApoC3* genotype					
S1S1	82	122	1		
S1S2 + S2S2	73	36	2.461	(1.545–3.919)	<0.01
Han ethnicity					
*ApoC3* genotype					
S1S1	83	117	1		
S1S2 + S2S2	78	37	2.899	(1.790–4.694)	<0.01

The model was adjusted for age, body mass index, smoking status and drinking status. OR: odds ratio, CI: confidence interval. S1S1 genotype versus S1S2 + S2S2 genotype in the Li and Han ethnic groups analyzed using multivariate unconditional logistic regression analysis

### Lipid profiles of atherosclerotic and healthy participants in the li and Han ethnic groups

FBS, TG, TC, LDL-C, apoB and apoCIII levels were significantly higher in atherosclerotic subjects compared to those in healthy controls, while HDL-C and apoAI levels and the apoAI:apoB ratio were significantly lower (all *P* < 0.01). There was no significant difference in Lp(a) levels (*P* > 0.05) between atherosclerotic patients and healthy controls (Table 6). Further analysis revealed differences in the lipid profiles of the Li and Han ethnic groups. Although increased TG, apoB, and apoCIII levels and decreased HDL-C levels were found in both ethnic groups, the apoAI levels and apoAI:apoB ratio were lower among atherosclerotic patients in the Li ethnic group (both *P* < 0.01) in comparison to those of Han descent, who typically showed increased TC and LDL-C levels and a high apoAI:apoB ratio (all *P* < 0.01) (Table 7).

**Table 6:** Lipid profiles of atherosclerotic patients (AS) and healthy controls (control)

Parameter	AS (n = 316)	Control (n = 312)	*t*	*P-*value
FBS (mmol /L)	5.88 ± 1.30**	5.67 ± 0.48	2.795	0.005
TC (mmol /L)	5.08 ± 1.10**	4.77 ± 0.80	4.087	0.000
TG (mmol/L)	1.44 ± 1.01**	1.15 ± 0.66	4.245	0.000
HDL-C (mmol/L)	1.12 ± 0.37**	1.27 ± 0.32	−5.443	0.000
LDL-C (mmol/L)	3.16 ± 0.90**	2.83 ± 0.73	4.984	0.000
ApoAI (g/L)	1.20 ± 0.36**	1.28 ± 0.34	−2.964	0.003
ApoB (g/L)	1.03 ± 0.23**	0.87 ± 0.18	9.418	0.000
ApoAI / ApoB	1.24 ± 0.50**	1.54 ± 0.53	−7.226	0.000
Lp(a) (mg/L)	250.02 ± 183.39	227.80 ± 168.16	1.583	0.114
ApoCIII (mg/dL)	16.36 ± 6.20**	10.95 ± 4.45	12.576	0.000

Data are expressed as mean ± SD. FBS: Fasting blood sugar; TC: Total cholesterol; TG: Triglyceride; HDL-C: High density lipoprotein cholesterol; LDL-C: Low density lipoprotein cholesterol; ApoAI: Lipoprotein AI; ApoB: Lipoprotein B; Lp(a): Lipoprotein a; ApoCIII: Apolipoprotein CIII; ^**^ *P* < 0.01, atherosclerotic patients versus healthy controls analyzed using *t-*tests

**Table 7:** Comparison of lipid profiles of atherosclerotic patients (AS) and healthy controls (control) in the Li and Han ethnic groups

Parameter	Li ethnicity			Han ethnicity		
	AS (*n* = 155)	Control (*n* = 158)	*P-*value	AS (*n* = 161)	Control (*n* = 154)	*P-*value
TC (mmol/L)	5.12 ± 1.29	4.99 ± 0.76	0.273	5.04 ± 0.89**	4.54 ± 0.77	0.000
TG (mmol/L)	1.34 ± 0.78*	1.18 ± 0.59	0.039	1.54 ± 1.18**	1.13 ± 0.73	0.000
HDL-C (mmol/L)	1.20 ± 0.40**	1.33 ± 0.29	0.002	1.04 ± 0.34**	1.21 ± 0.33	0.000
LDL-C (mmol/L)	3.10 ± 1.02	2.90 ± 0.73	0.058	3.22 ± 0.78**	2.76 ± 0.73	0.000
ApoAI (g/L)	1.28 ± 0.41**	1.39 ± 0.34	0.009	1.12 ± 0.29	1.17 ± 0.31	0.150
ApoB (g/L)	0.96 ± 0.24**	0.84 ± 0.16	0.000	1.11 ± 0.24**	0.89 ± 0.19	0.000
ApoAI/ApoB	1.40 ± 0.55**	1.70 ± 0.52	0.000	1.09 ± 0.21**	0.90 ± 0.19	0.000
Lp (a) (mg/L)	238.4 ± 161.4	225.3 ± 152.8	0.460	247.9 ± 176.6	232.8 ± 165.5	0.434
ApoCIII (mg/dL)	15.83 ± 6.12^**^	11.49 ± 4.51	0.000	16.87 ± 6.25**	10.39 ± 4.34	0.000

Data are expressed as mean ± SD. TC: Total cholesterol; TG: Triglyceride; HDL-C: High density lipoprotein cholesterol; LDL-C: Low density lipoprotein cholesterol; ApoAI: Lipoprotein AI; ApoB: Lipoprotein B; Lp(a): Lipoprotein a; ApoCIII: Apolipoprotein CIII; ^*^ *P* < 0.05, ^**^ *P* < 0.01, atherosclerotic patients versus healthy controls in the Li and Han ethnic groups analyzed using *t-*tests

### Lipid profiles of S1 and S2 allele carriers among the atherosclerotic patients and healthy controls in the li and Han ethnic groups

In the Li ethnic group, S2 allele carriers exhibited increased plasma apoCIII levels compared to those of S1 allele carriers among the atherosclerotic patients (*P* < 0.01), while the S2 allele carriers showed increased TG and apoCIII levels among the healthy controls (*P* < 0.01; *P* < 0.05) (Table 8). In the Han ethnic group, S2 allele carriers exhibited increased TG levels compared to S1 allele carriers among the atherosclerotic patients, while S2 allele carriers showed increased plasma apoCIII levels among the healthy controls (*P* < 0.01) (Table 9).

**Table 8:** Lipid profiles between S1 allele carriers and S2 allele carriers in the Li ethnic group

Parameter	AS (n = 155)			Control (n = 158)		
	S1S1 (*n* = 82)	S1S2 + S2S2 (*n* = 73)	*P*-value	S1S1 (*n* = 122)	S1S2 + S2S2 (*n* = 36)	*P*-value
TC (mmol/L)	5.12 ± 1.32	5.13 ± 1.29	0.956	4.95 ± 0.78	5.12 ± 0.72	0.259
TG (mmol/L)	1.26 ± 0.63	1.43 ± 0.92	0.166	1.07 ± 0.44	1.53 ± 0.84^**^	0.003
HDL-C (mmol/L)	1.26 ± 0.42	1.14 ± 0.36	0.064	1.33 ± 0.29	1.32 ± 0.29	0.982
LDL-C (mmol/L)	3.03 ± 0.99	3.17 ± 1.05	0.405	2.90 ± 0.72	2.91 ± 0.77	0.917
ApoAI (g/L)	1.29 ± 0.43	1.26 ± 0.39	0.644	1.41 ± 0.36	1.33 ± 0.26	0.243
ApoB (g/L)	0.94 ± 0.22	0.98 ± 0.26	0.260	0.85 ± 0.16	0.83 ± 0.15	0.610
ApoAI/ApoB	1.44 ± 0.58	1.36 ± 0.52	0.362	1.72 ± 0.53	1.66 ± 0.49	0.569
Lp(a) (mg/L)	254.0 ± 168.1	218.8 ± 156.3	0.181	231.5 ± 164.1	208.0 ± 101.3	0.298
ApoCIII (mg/dL)	14.07 ± 5.21	17.81 ± 6.48^**^	0.000	10.90 ± 3.96	13.53 ± 5.60^*^	0.012

Data are expressed as mean ± SD. TC: Total cholesterol; TG: Triglyceride; HDL-C: High density lipoprotein cholesterol; LDL-C: Low density lipoprotein cholesterol; ApoAI: Lipoprotein AI; ApoB: Lipoprotein B; Lp(a): Lipoprotein a; ApoCIII: Apolipoprotein CIII; ^*^ *P* < 0.05, ^**^ *P* < 0.01, S1 allele carriers versus S2 allele carriers in the Li ethnic group analyzed using the Kruskal–Wallis or Wilcoxon–Mann–Whitney tests

**Table 9:** Lipid profiles in S1 allele carriers and S2 allele carriers in the Han ethnic group

Parameter	AS (*n* = 151)			Control (n = 154)		
	S1S1 (*n* = 83)	S1S2 + S2S2 (*n* = 78)	*P*-value	S1S1 (*n* = 117)	S1S2 + S2S2 (*n* = 37)	*P*-value
TC (mmol/L)	4.92 ± 0.79	5.18 ± 0.97	0.065	4.52 ± 0.77	4.60 ± 0.78	0.622
TG (mmol/L)	1.27 ± 0.54	1.84 ± 1.56**	0.003	1.06 ± 0.53	1.36 ± 1.13	0.122
HDL-C (mmol/L)	1.08 ± 0.37	0.99 ± 0.29	0.117	1.23 ± 0.36	1.14 ± 0.25	0.089
LDL-C (mmol/L)	3.17 ± 0.73	3.28 ± 0.82	0.389	2.76 ± 0.74	2.77 ± 0.70	0.895
ApoAI (g/L)	1.11 ± 0.29	1.13 ± 0.29	0.682	1.15 ± 0.31	1.24 ± 0.29	0.097
ApoB (g/L)	1.07 ± 0.21	1.10 ± 0.21	0.266	0.97 ± 0.20	0.86 ± 0.17	0.291
ApoAI/Apo B	1.10 ± 0.42	1.10 ± 0.34	0.522	1.33 ± 0.52	1.48 ± 0.43	0.111
Lp(a) (mg/L)	244.6 ± 159.4	251.4 ± 194.2	0.806	222.3 ± 152.8	265.8 ± 199.3	0.164
ApoCIII (mg/dL)	15.97 ± 5.89	17.83 ± 6.51	0.059	9.78 ± 3.87	12.32 ± 5.15**	0.008

Data are expressed as mean ± SD. TC: Total cholesterol; TG: Triglyceride; HDL-C: High density lipoprotein cholesterol; LDL-C: Low density lipoprotein cholesterol; ApoAI: Lipoprotein AI; ApoB: Lipoprotein B; Lp(a): Lipoprotein a; ApoCIII: Apolipoprotein CIII; ^**^ *P* < 0.01, S1 allele carriers versus S2 allele carriers in the Han ethnic group analyzed using the Kruskal–Wallis or Wilcoxon–Mann–Whitney tests

## Discussion

Our study confirmed the existence of the *Sst*I polymorphism in the *apoC3* gene in the Li ethnic group, with increased frequencies of the S1S2 and S2S2 genotypes and the S2 allele found in atherosclerotic patients. Furthermore, the S2 allele was associated with an increased risk of atherosclerosis.

The S2 allele, unfavorable lipid profiles (increased FBS, TG, TC, LDL-C, apoB, and apoCIII; decreased HDL-C, apoAI and lower apoAI:apoB ratio) and environmental risk factors, such as elevated waist circumference and increased systolic and diastolic pressure were found to promote the development of atherosclerosis. We identified variations in these results when we investigated lipid profiles and the roles they played in atherosclerosis susceptibility in the Li and Han ethnic groups. According to our results, the S2 allele was consistently associated with plasma apoCIII in the Li population, while the association with plasma TG existed only in the healthy controls in this ethnic group. Based on the association of apoCIII with TG reported in a previous study [10, 15–17], we speculate that apoCIII might represent a link between the S2 allele and plasma TG levels, although this was not the only parameter which affected plasma TG, especially in the presence of atherosclerosis. In the Han population, the S2 allele was associated with increased plasma TG levels in the atherosclerotic group and with increased plasma apoCIII levels in the healthy controls. The variation in the association of the S2 allele with plasma TG or apoCIII levels indicated the importance of our study. The S2 allele and elevation of either plasma apoCIII or TG were found to be associated with the development of atherosclerosis in both the Li and Han ethnic groups. The *Sst*I polymorphism in the *apoC3* gene was also shown to influence the risk of atherosclerosis. In contrast, a recent study showed that non-mutated *apoC3* gene carriers exhibited a favorable lipid profile, and revealed strong genetic evidence implicating the S2 allele in the development of atherosclerosis [18, 19].

Our study revealed the association of the S2 allele with plasma apoCIII levels, which combined with unfavorable lipid profiles and environmental risk factors, contributed to the development of atherosclerosis in the Li ethnic group. Thus, it can be hypothesized that ApoCIII functions as a link between the S2 allele and plasma TG levels, although the exact mechanism of the interactions among the S2 allele and the levels of plasma apoCIII and TG remain to be clarified.

## Conclusions

The association of the S2 allele of the *Sst*I polymorphism in the *apoC3* gene with plasma apoCIII levels interacts with unfavorable lipid profiles to contribute to the development of atherosclerosis in the Li population in China.

## Abbreviations

ApoAI: Lipoprotein AI
ApoB: Lipoprotein B
ApoCIII: Apolipoprotein CIII
BMI: Body mass index
CHD: Coronary heart disease
DNA: Deoxyribonucleic acid
EDTA: Ethylene diamine tetra-acetic acid
ELISA: Enzyme-linked immunosorbent assay
FBS: Fasting blood sugar
HDL-C: High density lipoprotein cholesterol
IDL: Intermediate density lipoprotein
LDL: Low density lipoprotein
LDL-C: Low density lipoprotein cholesterol
Lp(a): Lipoprotein a
PCR: Polymerase chain reaction
RFLP: Restriction fragment length polymorphism
TC: Total cholesterol
TG: Triglyceride
THP-1: Human acute monocytic leukemia cell line
VLDL: Very low density lipoprotein
